# Public health round-up

**DOI:** 10.2471/BLT.21.011221

**Published:** 2021-12-01

**Measles vaccination catch-up**

Measles vaccination supplies being prepared for transportation from the city of Herat to Ghor province in Afghanistan on 19 September 2021. Afghanistan is one of many countries to suffer measles outbreaks as a result of pandemic-related low routine vaccination coverage (a problem exacerbated in Afghanistan by recent political instability). According to a new report, worldwide, more than 22 million infants missed their first dose of measles vaccine in 2020, three million more than in 2019, raising concerns about increased risk of measles outbreaks.

**Figure:**
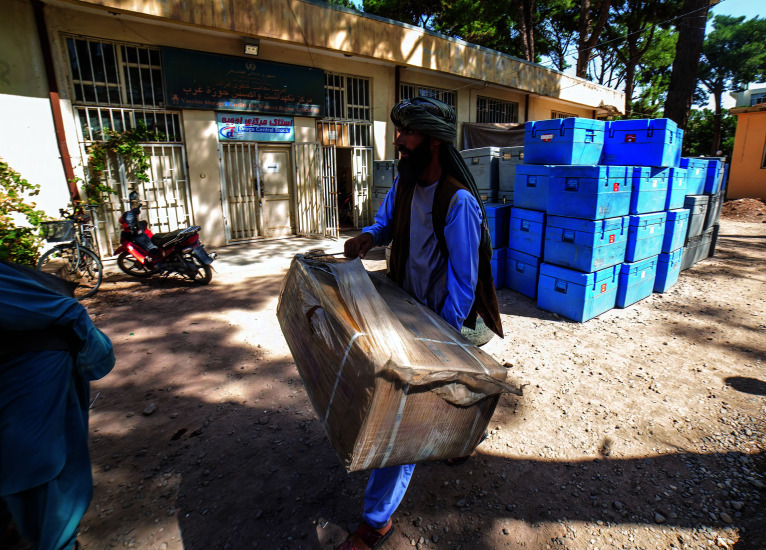
UNICEF/Sayed Bidel

## WHO validates new COVID-19 vaccine

The World Health Organization (WHO) issued an emergency use listing (EUL) for the coronavirus disease 2019 (COVID-19) vaccine COVAXIN on 3 November. Derived from an inactivated severe acute respiratory syndrome coronavirus 2 (SARS-CoV-2) antigen, COVAXIN was found to be 78% effective against COVID-19 of any severity, 14 or more days after the second dose. The vaccine can be stored at 2–8°C.

WHO’s Strategic Advisory Group of Experts on Immunization, which formulates vaccine-specific policies and recommendations for vaccines’ use in populations, recommended use of the vaccine in two doses, with a dose interval of four weeks, in all age groups 18 and above.

COVAXIN was developed by Bharat Biotech (an Indian multinational biotech company) in collaboration with the Indian Council of Medical Research National Institute of Virology.

EUL is a prerequisite for inclusion in the COVAX vaccine supply mechanism. It also allows countries to expedite their own regulatory approval to import and administer COVID-19 vaccines.

https://bit.ly/3wOLZdL

## Measles progress threatened

More than 22 million infants missed their first dose of measles vaccine in 2020, three million more than in 2019, raising concerns about increased risk of measles outbreaks, going forward. This is according to *Progress toward regional measles elimination – worldwide, 2000–2020*, a new report from WHO and the United States of America’s Centers for Disease Control and Prevention which was released on 10 November.

The COVID-19 pandemic was a key factor in the vaccination shortfall, with 24 measles vaccination campaigns in 23 countries originally planned for 2020 being postponed as a result of pandemic-related challenges, including reallocation of resources and social distancing measures. Measles surveillance also deteriorated. This may explain the dramatic fall in reported measles incidence, which dropped from 120 to 22 per million of population between 2019 and 2020.

“While reported measles cases dropped in 2020, evidence suggests we are likely seeing the calm before the storm as the risk of outbreaks continues to grow around the world,” said Dr Kate O’Brien, Director of WHO’s Department of Immunization, Vaccines and Biologicals.

O’Brien called for countries to vaccinate as quickly as possible against COVID-19 but stressed that this must not come at the cost of essential immunization programmes. “Routine immunization must be protected and strengthened; otherwise, we risk trading one deadly disease for another,” she said.

https://bit.ly/3oB7KKn

## Solidarity Trial Vaccines

WHO and the health ministries of Colombia, Mali and the Philippines announced the launch of the co-sponsored Solidarity Trial Vaccines, an international, randomized clinical trial that aims to accelerate the evaluation of promising candidate COVID-19 vaccines, contributing to the creation of a larger portfolio of vaccines needed to protect people from COVID-19 around the world.

Announced 26 October, the trial also has the potential to uncover second-generation vaccines that may confer longer protection against SARS-CoV-2 variants of concern, or be more convenient to administer, for example through oral formulations.

The trial is beginning with research teams in over 40 trial sites spread across the three countries and will initially focus on two candidate vaccines – a protein subunit vaccine and a DNA vaccine coding for the SARS-CoV-2 spike protein. Two additional vaccines are expected to enter the trial once additional evidence and documentation has been reviewed.

https://bit.ly/3wNWsGj

## Climate smart health commitments

A group of 50 countries committed to developing climate-resilient and low-carbon health systems at the United Nations Climate Change Conference in Glasgow (COP26), in response to growing evidence of the impact of climate change on people’s health.

Announced on 9 November, the commitments were made as part of the COP26 Health Programme, a partnership between the government of the United Kingdom of Great Britain and Northern Ireland, WHO, the United Nations Framework Convention on Climate Change Climate Champions and nongovernmental organizations.

“We applaud those countries that have committed to building climate-resilient and low-carbon health systems, and we hope to see many others following their lead in the near future,” said WHO Director-General Tedros Adhanom Ghebreyesus.

https://bit.ly/3C7jitt

## Group B streptococcus impact

New research published by WHO and the London School of Hygiene & Tropical Medicine on 2 November sharpens the focus on the global disease burden of Group B streptococcus (GBS), quantifying for the first time its contribution to preterm births, as well as to neurological impairments that can occur following GBS-associated infections.

Although the bacterium is harmless for most pregnant women who carry it, it can be serious when it passes to babies during pregnancy, childbirth or in the early weeks of life. Linked to nearly 100 000 newborn deaths and at least 46 000 stillbirths each year, infection with the bacterium is also associated with long-term disability, and with over half a million preterm births.

New vaccines are urgently needed to reduce the death and disease associated with the pathogen. Potential vaccines have been in the development pipeline for three decades.

https://bit.ly/3C5zdZ2

## Insulin access challenges

The state of global access to insulin and diabetes care was set out in a new report published by WHO in the lead-up to World Diabetes Day. Released on 12 November, the report underlines ongoing challenges to universal access that include the lack of competition in a market that is dominated by three multinational companies, the shift towards higher-priced synthetic insulins known as insulin analogues in the global market, the lack of transparency in the way prices are set, and suboptimal government regulation and policies.

Insulin is the bedrock of diabetes treatment – for people with both type 1 and type 2 diabetes. One out of every two people needing insulin for type 2 diabetes does not get it.

The report outlines recent commitments made by insulin manufacturers and suggests several actions to improve access to insulin and related products.

https://bit.ly/3wMhncu

**Cover photo**

Students during a surf rescue course at Hojo Beach in Tateyama, Chiba Prefecture, Japan, 26 June 2021, as part of the Japan Lifesaving Association’s work to educate new licensed lifesavers and prevent drowning in the country.

**Figure:**
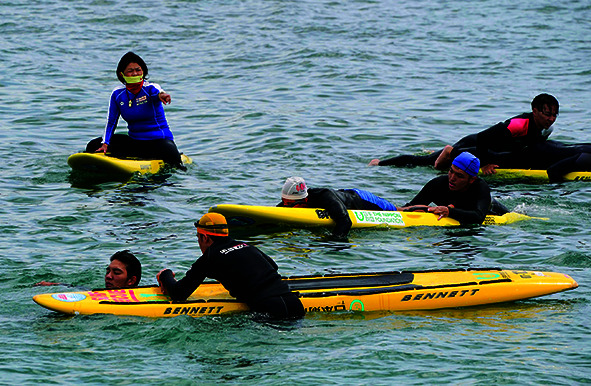
WHO/Kimimasa Miyama

## Financing Health for All

WHO’s Council on the Economics of Health for All called for clear, ambitious goals to be set to reform and redirect health financing to achieve the objective of Health for All.

In its quarterly brief, *Financing health for all*, released on 26 October ahead of the G20 Summit in Rome, Italy, the Council frames financing for health as a long-term investment rather than a short-term cost, and highlights the need to increase and optimize health financing by creating greater fiscal space, re-directing investment, and introducing regulatory and financing measures that augment and direct private finance with a view to achieving equitable improvements in health outcomes.

“While health systems are under-resourced, more finance is not the only solution,” said Professor Mariana Mazzucato, Chair of the Council, stressing the need to reform and redirect health finance so that the objective of Health for All is “designed into” the financial structures, the conditionalities and the partnerships between business and the state. 

https://bit.ly/30tDXuU

## Global tobacco use down

The number of tobacco users is estimated to have fallen by 20 million since 2015, down from 1.32 billion to 1.30 billion today. A further decline to 1.27 billion is expected by 2025.  

This is according to the fourth *WHO global tobacco trends report* which was released on 16 November. The report notes that sixty countries are now on track to achieving a voluntary global target of a 30% reduction in tobacco use between 2010 and 2025, up from only 32 countries two years ago. 

The report credits effective, comprehensive tobacco control policies under the WHO Framework Convention on Tobacco Control (WHO FCTC) and MPOWER with driving the trends. Trends in use of electronic cigarettes and other nicotine delivery devices are not included in the report because there are not yet enough country data to make global or regional estimates.

https://bit.ly/3CizkAM

**Looking ahead**

7–8 December. Nutrition for Growth Summit. https://bit.ly/3mKhNgr

6–11 December. International Conference on AIDS and Sexually Transmitted Infections in Africa. https://bit.ly/3qEBPLG

13–15 December, 10th Global Conference on Health Promotion. https://bit.ly/3EK0q5h

